# Measurement of T_1_ and T_2_ relaxation times of the pancreas at 7 T using a multi-transmit system

**DOI:** 10.1007/s10334-019-00768-w

**Published:** 2019-07-17

**Authors:** Mariska Damen, Maarten van Leeuwen, Andrew Webb, Dennis Klomp, Catalina Arteaga de Castro

**Affiliations:** 1 Radiology, Leiden University Medical Center, Leiden, The Netherlands; 2 Radiology, University Medical Center Utrecht, Utrecht, The Netherlands; 3 Imaging Division, University Medical Center Utrecht, Utrecht, The Netherlands

**Keywords:** Pancreas, T_1_ relaxation, T_2_ relaxation, High field, 7 T

## Abstract

**Objective:**

To determine T_1_ and T_2_ relaxation times of healthy pancreas parenchyma at 7 T using a multi-transmit system.

**Materials and methods:**

Twenty-six healthy subjects were scanned with a 7 T MR system using eight parallel transceiver antennas, each with two additional receive loops. A Look-Locker sequence was used to obtain images for T_1_ determination, while T_2_ was obtained from spin-echo images and magnetic resonance spectroscopy measurements with different echo times. T_1_ and T_2_ times were calculated using a mono-exponential fit of the average magnitude signal from a region of interest in the pancreas and were tested for correlation with age.

**Results:**

The age range of the included subjects was 21–72 years. Average T_1_ and T_2_ relaxation times in healthy pancreas were 896 ± 149 ms, and 26.7 ± 5.3 ms, respectively. No correlation with age was found.

**Conclusion:**

T_1_ and T_2_ relaxation times of the healthy pancreas were reported for 7 T, which can be used for image acquisition optimization. No significant correlations were found between age and T_1_ or T_2_ relaxation times of the pancreas. Considering their low standard deviation and no observable age dependence, these values may be used as a baseline to study potentially pancreatic tissue affected by disease.

## Introduction

Magnetic resonance (MR) imaging is an imaging modality with good soft tissue contrast and high sensitivity for detection of pancreatic cancer, which facilitates early tumor detection [1, 2]. Modern MRI techniques can visualize biliary and pancreatic ductal systems noninvasively, have a high sensitivity for tumor detection, and, unlike endo-ultrasound imaging (EUS) with high sensitivity (up to 89%) and specificity (up to 99%) for detection of small-tumor pancreatic cancer [3–5], reveal the pancreatic three-dimensional anatomy, possible invasion into surrounding tissue or vascular involvement [6]. However, it is still difficult to distinguish chronic pancreatitis and pancreatic carcinoma using T_1_-weighted and/or T_2_-weighted MR images on 1.5 and 3 Tesla (T) [7], even when gadolinium contrast agents are used [8].

Higher magnetic field strengths, such as 7 T, offer higher signal-to-noise (SNR) and contrast-to-noise ratios (CNR), as well as higher spatial and spectral resolutions [9]. However, artifacts are more profound at higher field strengths, particularly in the abdominal region, mainly due to the reduced wavelength in the body that leads to an inhomogeneous B_1_^+^ field distribution and signal voids [10, 11]. In addition, the local specific absorption rate (SAR) increases with increasing field strength [12], leading to longer repetition times needed and thus longer acquisitions. Using a multi-transmit system, the B_1_^+^ efficiency can be optimized within a region of interest (ROI). This can be achieved by optimizing the transmit phases for each channel in the body coil. As a result, improved local B_1_^+^ homogeneity and magnitude can be achieved [10].

In addition to the technical challenges that MRI at higher magnetic field strengths brings, high-quality diagnostic imaging of pancreatic cancer requires development and optimization of imaging protocols. Moreover, a new baseline assessment of the images obtained at ultra-high field is required. Therefore, knowledge of relaxation times (T_1_ and T_2_) is essential. These characteristics are known to change substantially with field strength and this change cannot be predicted accurately with theoretical calculations due to the complex tissue behavior [13]. Consequently, although T_1_ and T_2_ values have been well established for many tissues, including pancreatic tissue at 1.5 T and 3 T [14–16], T_1_ and T_2_ relaxation times of pancreatic tissue at 7 T remain unknown.

The purpose of this study was to determine the mean T_1_ and T_2_ relaxation times of healthy pancreas parenchyma over a wide age range at 7 T to assess baseline levels to develop optimized imaging protocols at this magnetic field strength.

## Materials and methods

### Study population and hardware

Twenty-six healthy subjects were scanned with a 7 T MR system (Philips, Best, The Netherlands) after providing a written informed consent. A multi-transmit system with eight parallel transmit channels was used, where each channel was connected to a transmit-receive fractionated dipole antenna (MR Coils BV, Drunen, The Netherlands) [17]. Each antenna had 2 additional receive loops integrated in its housing (16 in total) and were interfaced to a 16-channel receiver box (Philips, Best, The Netherlands).

### Image acquisition

All scans were acquired in a ‘feet first’ supine position, with the eight channels positioned symmetrically around the abdomen, approximately centered at the height of the pancreas. First, a gradient echo image was obtained for anatomy localization and optimization steps (2D T1 fast field echo (FFE), FA = 15°, TR/TE = 10/5 ms, FOV = 704 × 704 mm^2^, 0.7 × 0.7 × 10 mm^3^ voxels). Part of the optimization was RF phase shimming, in which the RF phase of each antenna is steered to maximize and homogenize the B_1_^+^ field in the pancreas region (ROI). An in-house developed MATLAB (^©^MATLAB 2015b) script was used for loading a dynamic imaging series (fast field echo (FFE), FA = 4°, TR/TE = 30/1.68 ms, 0:25 min total acquisition time, 1.1 × 1.1 × 10 mm^3^ voxels), drawing an ROI including the whole pancreas, and optimizing the phase of each element, while keeping a fixed power using a numerical optimization algorithm. After this, image-based B_0_ shimming was performed. Subsequently, a B_1_^+^ map was acquired (fast field echo (FFE), FA = 50°, TR/TE = 50–250/2.2 ms, 1:29 min total acquisition time, 1.2 × 1.2 × 10 mm^3^ voxels) for power optimization in the pancreas region. Finally, to guarantee optimal performance in the T_2_-weighted MRI protocol, the power was adjusted to reach a B_1_^+^ of 10 µT within the pancreas.

A Look-Locker sequence was used for T_1_ relaxation determination in one slice containing the pancreas in 22 subjects (inversion recovery turbo field echo (TFE), FA = 3°, TR/TE = 40/1.68 ms, 20 measurement times separated by 100 ms, during a 6 s cycle, 8:33 min total acquisition time, 4 × 4 × 10 mm^3^ voxels).

**Fig. 1:**
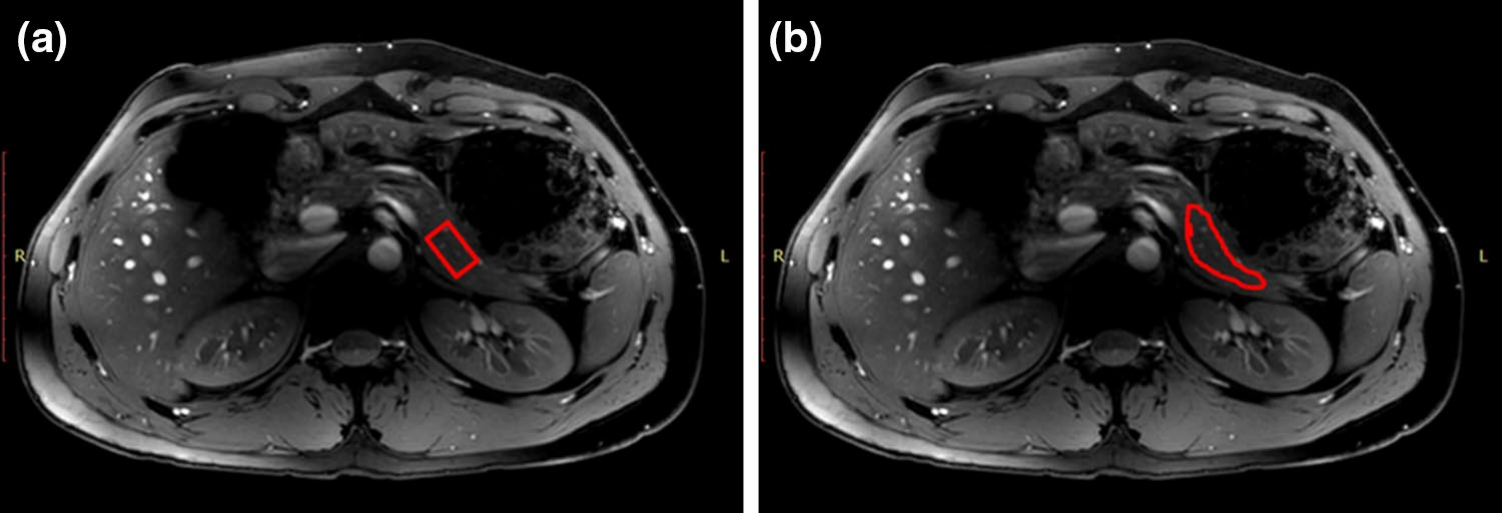
Examples of ROIs (in red) drawn in pancreas for **a** STEAM spectroscopy and **b** T_2_-weighted imaging series acquired at different echo times

Since T_2_ relaxation time quantification is known to be sequence dependent [18–20], we used three different methods to quantify the T_2_ relaxation times. In the first method (1), including three subjects, separated T_2_-weighted images were acquired in a single slice for four different echo times to determine T_2_ relaxation times in the pancreas using a 2D single-shot turbo spin-echo (2D single-shot turbo spin-echo (TSE), effective TR/TE = 20 s/50; 80; 100; 150 ms, refocusing FA = 180°, mean pixel BW = 341 Hz/px, 1.5 × 1.5 × 5 mm^3^ voxels, FOV = 320 × 400 mm^2^, SAR = 1.2 W/kg, Acq. time per image = 20 s). The choice of this long effective TR was made to keep SAR within (conservative) safe limits. In the second method (2), including 4 subjects, magnetic resonance spectroscopy measurements were obtained with a stimulated echo acquisition mode (STEAM) sequence at 10 different echo times on a single voxel (Fig. 1a) placed in the pancreas (TEs = 20–110 ms, TM/TR = 1.22/1500 ms, 20 × 40 × 20 mm^3^ voxel, 2048 points, 4 kHz bandwidth, NSA = 4). Finally, the last method (3) was applied in the remaining subjects (16) to examine the age dependency of T_2_ relaxation times. This was done using a 2D single-shot turbo spin-echo acquired in a single slice separately for all different echo times with a refocusing pulse of 35° (2D single-shot turbo spin-echo (TSE), TR/TE = 10 s/ 50; 80; 100; 150 ms (TE_equiv_ = 24; 32; 36; 46 ms), FA 90°, mean pixel BW = 329 Hz/px, 1.5 × 1.5x5 mm^3^ voxels, FOV = 320 × 400 mm^2^, SAR = 0.15 W/kg, Acq. time per image = 10 s, TSE factor varying between 115 and 157, SPAIR fat suppression), which is a robust protocol with shorter scan times and lower SAR levels ( ~ 85% lower) when compared to the spin-echo sequence with a 180° refocusing angle described in method (1).

### Data processing

Mono-exponential decay with a non-linear least squares method was used to fit T_2_ relaxation times [21]; for the T_2_-weighted images magnitude signals were used and for the STEAM data the area under the curve of the water peak was used. To fit the T_2_ relaxation time from the lower refocusing angle series, the equivalent echo times (TE_equiv_) were used. This TE_equiv_ was the calculated apparent echo time (based on the average T_1_ and T_2_ relaxation times determined with the 180° refocusing pulse and STEAM) as if a 180° refocusing angle had been used [22]. An ROI was manually drawn within the area with the best B_1_^+^ homogeneity and B_1_^+^ closest to 100%, excluding big vessels (Fig. 1b). The position of the ROI in the pancreas was consistent within subjects, however, between subjects the location of the ROI varied between head, body and tail of the pancreas. The signal in the ROIs was subsequently averaged and fitted. Average T_1_ and T_2_ relaxation times were calculated. Finally, age dependency was examined using all three datasets.

### Statistical analysis

An unpaired *t* test was performed to compare T_2_ relaxation fitting methods (1) and (2). Since previously published research has shown a significant linear dependence of age with T_1_ relaxation times [23] and it is known that the pancreas tissue composition is changing with age [16], a linear regression analysis for age with respect to T_1_ and T_2_ was performed in SPSS (IBM Corp. Released 2015. IBM SPSS Statistics for Windows, Version 23.0. Armonk, NY: IBM Corp.).

## Results

Out of 26 subjects, 22 T_1_ and 23 T_2_ datasets could be used for further analysis. The discarded datasets had insufficient image quality, caused by motion or not enough B_1_^+^ amplitude in the pancreas region. Average age of the subjects was 37 years (range 21–72 years) for the T_1_ datasets and 40 years (range 24–72 years) for the T_2_ datasets.

**Fig. 2:**
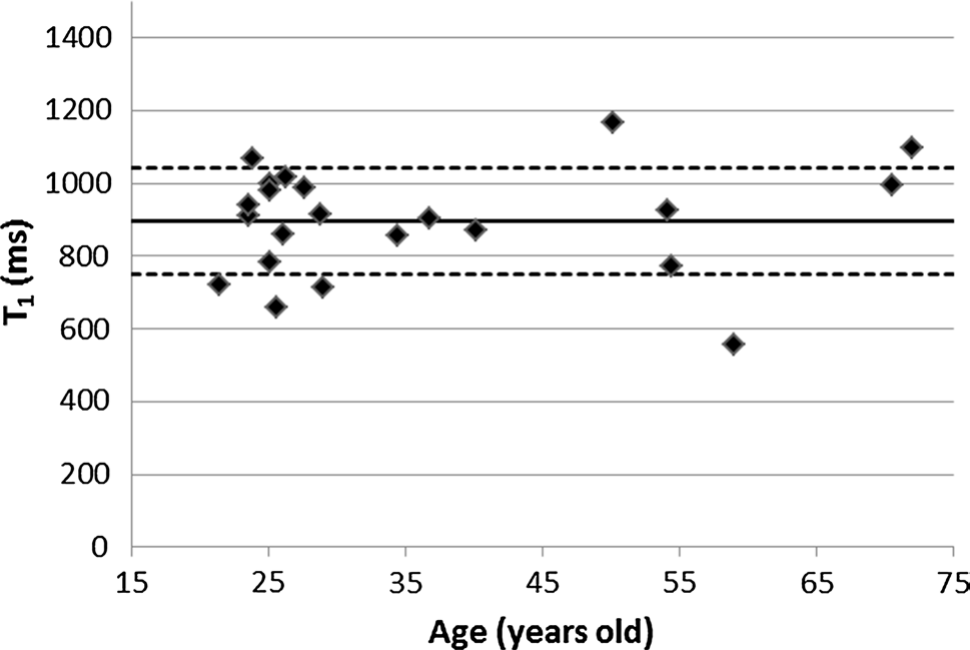
T_1_ relaxation times in a ROI in the pancreas for all individual subjects. The average T_1_ relaxation time was 896 ms with a standard deviation of 149 ms. The solid line indicates the average T_1_ relaxation time and the dashed lines indicate the mean ± the standard deviation

**Fig. 3:**
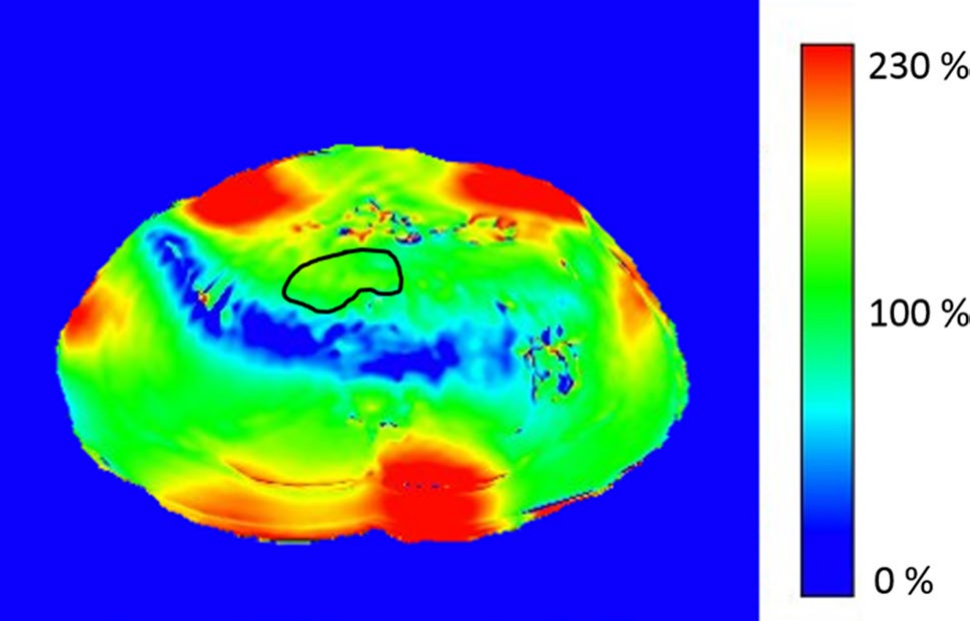
Example of a B_1_^+^ map acquired after B_1_ shimming of the pancreas for a single subject. The image scaling represents the measured B_1_^+^ in percent from the aimed B_1_^+^ of 10 µT. ROI in black showing the region used for data analysis, an average B_1_^+^ of 100% is achieved

T_1_ relaxation times (mean ± std) for all 22 subjects are summarized in Fig. 2, the average T_1_ relaxation time was 896 ± 149 ms. An example of an B_1_^+^ map is shown in Fig. 3, the mean ratio of the measured B_1_^+^ over the expected B_1_^+^ value in the ROI across the subjects was 0.92 ± 0.14. Linear regression analysis showed no correlation between age and T_1_ relaxation time (slope = 1.1, offset = 856, *R*^2^ = 0.01, *p* = 0.6).

Using method (1) (T_2_-weighted image series with a 180° refocusing pulse) and (2) (using STEAM spectroscopy) for T_2_ determinations gave an average T_2_ relaxation time of 28.1 ms (*n* = 3) and 25.6 (*n* = 4), respectively. An unpaired *t* test proved no significant difference (*p* = 0.6) between the results of the two methods. Combining these results as a reference value gave an average T_2_ of 26.7 ± 5.3 ms.

**Fig. 4:**
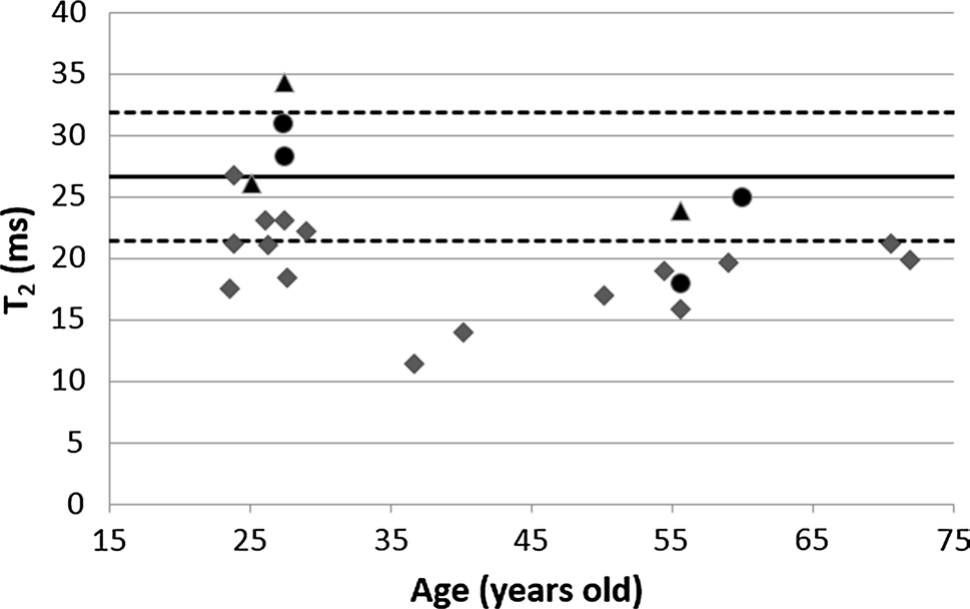
T_2_ relaxation times in a ROI in the pancreas for all individual subjects. The average T_2_ relaxation time was 26.7 ms with a standard deviation of 5.3 ms. Triangles show the individual subjects measured with method (1) (T_2_-weighted image series with a refocusing pulse of 180°), circles show method (2) (STEAM spectroscopy) and the squares show method (3) (T_2_-weighted image series with a refocusing pulse of 35°). The solid line indicates the average T_2_ relaxation time from methods (1) and (2) and the dashed lines indicate the mean ± the standard deviation

**Fig. 5:**
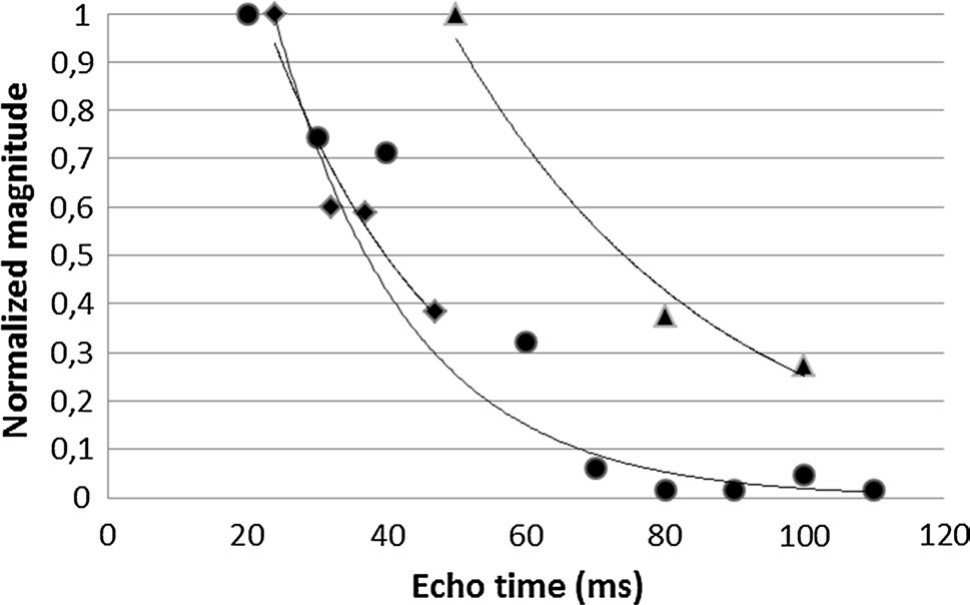
Individual T_2_ mono-exponential fits for all three methods for a single subject. Correspondence of the slope of the mono-exponential fits is shown. T_2_ relaxation times determined for these individual subjects using method (1)—T_2_-weighted image echo-series with 180° (triangles), method (2)—STEAM spectroscopy (circles) and method (3) 35° refocusing pulses (squares) were 34.3 ms, 28.3 ms and 23.1 ms, respectively

Method (3) resulted in a lower average T_2_ relaxation time of 19.5 ± 3.8 ms (*n* = 16) as expected due to the lower refocusing angle used. Fitted T_2_ relaxation times for all 23 subjects are summarized in Fig. 4. No correlation between age and T_2_ relaxation time was found (slope = − 0.1, offset = 31, *R*^2^ = 0.2, *p* = 0.07). Figure 5 shows examples of the mono-exponential fits for all three methods, with the signal of the first acquisition normalized to 1, for three single subjects.

**Fig. 6:**
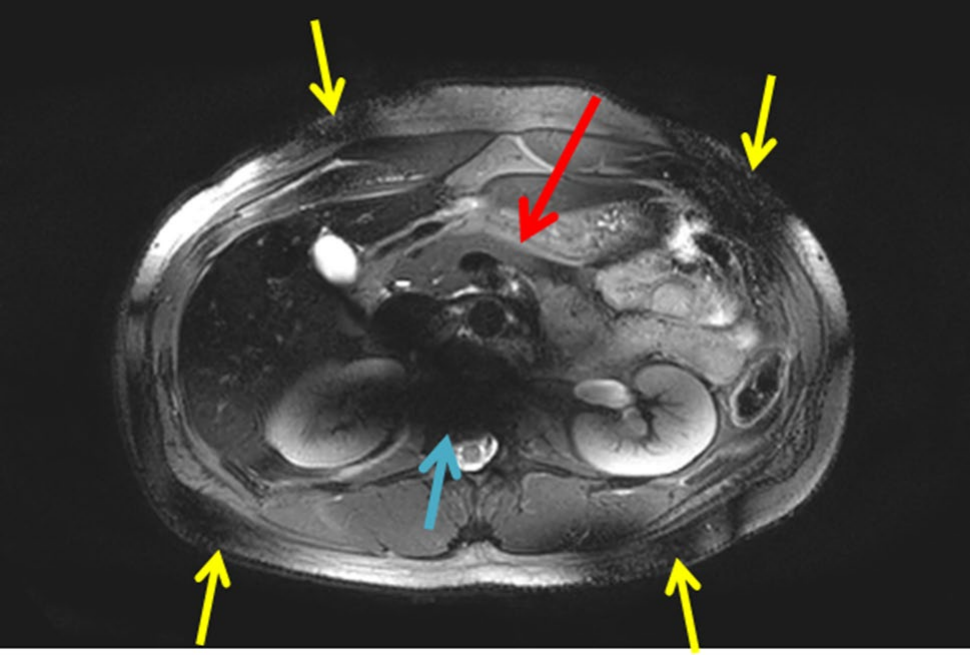
T_2_-weighted image for one subject using an equivalent echo time of 31 ms. 1.5 × 1.5 × 5 mm^3^ voxels. The red arrow points at the pancreas. Field inhomogeneities with signal voids outside the pancreas (blue arrow) can be observed. The yellow arrows point at regions where overtipping occurs (~ 180°) due to B_1_^+^ field inhomogeneity effects, leading to signal voids

Multi 2D T_2_-weighted single-shot turbo spin-echo images FA 90°, refocusing flip angle 35°, TE 80 ms (TE_equiv_ = 31 ms), TR 10 s, TSE factor of 121, SPAIR fat suppression, voxel size of 1.5 × 1.5 × 5 mm^3^ (Fig. 6) were obtained. TE_equiv_ close to the average T_2_ relaxation time of pancreas and TR more than 5 times longer ( ~ 11 times longer) than the average T_1_ relaxation time for improved contrast. The full field of view of the pancreas can be covered. However, signal voids can be still observed outside the region of interest (not B_1_^+^ optimized region), which are more pronounced in spin-echo-based sequences.

## Discussion

In this study, we determined normal pancreas T_1_ and T_2_ relaxation times in 26 healthy subjects aged 21–72 years at 7 T. T_1_ and T_2_ relaxation times for pancreas at 7 T—to our knowledge—have not been reported before. However, they have been reported for pancreas in healthy subjects at 1.5 T and 3 T. In the literature, it can be found that T_1_ relaxation times increase and T_2_ relaxation times decrease with increasing field strengths [13, 23]. De Bazelaire et al. [13] used an inversion recovery method and different inversion times for T_1_ measurements and a multiple spin-echo (SE) technique with different echo times for T_2_ measurements in six healthy subjects, reported T_1_ values of 584 ± 14 ms for 1.5 T and 725 ± 71 ms for 3 T and T_2_ values of 46 ± 6 ms for 1.5 T and 43 ± 7 ms for 3 T, and concluded that pancreas T_1_ relaxation times increased and T_2_ relaxation times decreased with increasing field strength. Tirkes et al. [23] reported a mean T_1_ relaxation time of 797 ms at 3 T for healthy pancreas (*n* = 53, sequence: dual flip angle 3D gradient echo). A slightly higher mean T_1_ of 987 ± 52 ms and mean T_2_ of 50 ± 3 ms at 3 T was reported by Chhor et al. [15] (*n *= 6, sequence: Look-Locker for T_1_ and T_2_-prep for T_2_ measurements). Our 7 T results confirm the expected trend of increasing T_1_ and decreasing T_2_ relaxation times with age.

In addition, T_1_ and T_2_ values are dependent on tissue composition [24]. In this study, we did not investigate tissue differences between subjects. Therefore, we could not confirm this at 7 T. Tirkes et al. [23] also found a weak correlation between age and T_1_ relaxation time in the pancreas at 3 T, which we could not observe in our measurements at 7 T. The relatively young mean age of them could explain the lack of correlation (if any).

The use of standard TSE sequences attenuates the effect of molecular diffusion by the use of multiple refocusing pulses, the effect becoming stronger as the refocusing pulses get closer to each other [14, 24]. In addition, differences in imaging methods and determining T_2_ relaxation times, a more than doubled magnetic field strength, different age ranges, and the morphology of each subject, combined with the complex characteristics of tissues, may be responsible for the large decrease of T_2_ relaxation time found at 7 T.

For T_1_ measurements, a Look-Locker sequence is generally used with a TR (including the 6 s per cycle) of more than 5 times the T_1_ value of pancreas tissue, which is required to measure the T_1_ value accurately. Using T_2_-weighted imaging with full 180° refocusing angle and magnitude signals to fit the water peaks from the MR spectroscopy measurements, T_2_ relaxation times for pancreas could be correctly determined. A spin-echo sequence such as the one used here in method (1) is not practical to use in such large groups of subjects. The high SAR deposition in the body due to the large refocusing angles requires long repetition times leading to long scanning times, to keep SAR under the guideline levels. Since the mentioned SAR levels was the worst-case possible SAR and it is known that the dipole array will never exceed a local SAR of 10 W/kg when driven at a time averaged power of 3 W per channel driving all eight antennas at any phase setting, the SAR could be overestimated but were always within (conservative) limits. In addition, there are hardware limitations associated to these high-power demanding sequences. T_2_ imaging with a small refocusing pulse ensures a more practical sequence; with shorter scan times and lower SAR levels. However, T_2_ imaging with a 35° refocusing pulse is affected by T_1_-weighted stimulated echoes during the signal formation [25], which leads to a lengthening of the signal-intensity decay. This resulted in longer T_2_ relaxation times, which after correction with the equivalent echo times (to a 180° refocusing angle), resulted in slightly lower T_2_ relaxation values and a comparable variability to the results found with the nominal 180° refocusing pulse and the spectroscopy data, as was seen in Fig. 4. No other explanation could be found for the underestimation of the results of method (3). Age-dependency testing using data from method (3) is, therefore, only suitable for assessing the relative differences of T_2_ of the pancreas with respect to age.

Inter-subject variability in the measured relaxation times can be caused by different factors. Variations within characteristics of (pancreatic) tissue, and their change with age were different for individuals, even between men and women (which we did not take into account). As Sato et al. [16] describes in their paper, the pancreas changes with increasing age; characteristic changes with age are pancreatic atrophy, lobulation and fatty degeneration. These changes, such as the fraction of fat in the pancreas would lead to altered relaxation times.

Results were fitted over scans with different echo times since a single sequence with multiple echo times given by the turbo factor resulted to be too sensitive to motion and breathing artifacts that would result in blurring of the image. This leads to slight differences in anatomy position between echo times. Therefore, the delineated ROIs did not correspond to exactly the same anatomical position, leading to outliers and a higher variation in the raw data, which can influence the fitting. In our study, data were obtained from all regions in the pancreas. Since the ROIs were drawn in the area with the best B_1_^+^ homogeneity, the location was different between subjects. Tirkes et al. [23] showed a slight but not significant difference between the relaxation times measured in head, body or tail. As we have not accounted for these differences, this might have been another factor affecting the standard deviation in our calculations.

Lastly, imaging the abdomen at higher field, especially with T_2_-weighted sequences, is challenging. The scans are more sensitive to artifacts given the increased B_0_ and B_1_^+^ inhomogeneities (even with good B_0_ and B_1_^+^ shimming) found at higher field strengths, leading to greater standard deviations sources.

The measured T_1_ and T_2_ relaxation times for pancreas at 7 T reported here is a starting point for pancreatic research at higher field strengths to improve abdominal MR imaging, and may be a base for further use of MR techniques to noninvasive pancreatic diagnostics particularly in early stages of disease.

## Conclusion

T_1_ and T_2_ relaxation times of the healthy pancreas were reported for 7 T, which can be used for image acquisition optimization. No significant correlation was found between age and T_1_ or T_2_ relaxation times of the pancreas.
